# Supplementary material for: A bacterial assay for rapid screening of IAA catabolic enzymes
Source: Plant Methods. 2019 Nov 4;15:126. doi: 10.1186/s13007-019-0509-6 (PMC6827244; doi:10.1186/s13007-019-0509-6)
Supplement: Supplementary file 4 — Additional file 4: Figure S2. Analysis of IAA and oxIAA background concentrations in liquid media containing AtDAO1- or GFP-producing bacteria or no bacteria prior supplementation with exogenous IAA (T0). Mean ± SD (n = 3). [file 13007_2019_509_MOESM4_ESM.pdf]

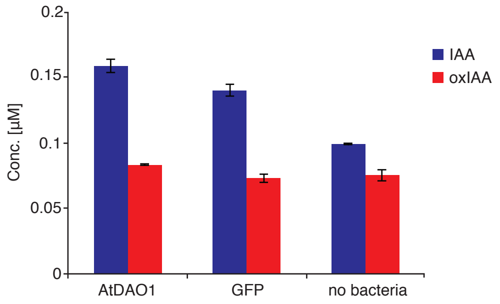

Figure S2. Analysis of IAA and oxIAA background concentrations in liquid media containing AtDAO1- or GFP-producing bacteria or no bacteria prior supplementation with exogenous IAA (T0). Mean ± SD (*n*=*3*).
